# An Interface–Particle Interaction Approach for Evaluation of the Co-Encapsulation Efficiency of Cells in a Flow-Focusing Droplet Generator

**DOI:** 10.3390/s20133774

**Published:** 2020-07-05

**Authors:** Mohammad Yaghoobi, Mohammad Said Saidi, Sepehr Ghadami, Navid Kashaninejad

**Affiliations:** 1 Department of Mechanical Engineering, Sharif University of Technology, Azadi St., Tehran 11155, Iran; mhmd.jcb1993@gmail.com; 2 Department of Mechanical Engineering, University of Waterloo, 200 University Avenue West, N2L 3G, Waterloo, ON N2L 3G1, Canada; sghadami@uwaterloo.ca; 3 Queensland Micro- and Nanotechnology Centre, Nathan Campus, Griffith University, 170 Kessels Road, Brisbane QLD 4111, Australia

**Keywords:** finite element method, droplet generator, microfluidics, encapsulation efficiency, flow focusing, particle interaction

## Abstract

Droplet-based microfluidics offers significant advantages, such as high throughput and scalability, making platforms based on this technology ideal candidates for point-of-care (POC) testing and clinical diagnosis. However, the efficiency of co-encapsulation in droplets is suboptimal, limiting the applicability of such platforms for the biosensing applications. The homogeneity of the bioanalytes in the droplets is an unsolved problem. While there is extensive literature on the experimental setups and active methods used to increase the efficiency of such platforms, passive techniques have received less attention, and their fundamentals have not been fully explored. Here, we develop a novel passive technique for investigating cell encapsulation using the finite element method (FEM). The level set method was used to track the interfaces of forming droplets. The effects of walls and the droplet interfaces on relatively large cells were calculated to track them more accurately during encapsulation. The static surface tension force was used to account for the effects of the interfaces on cells. The results revealed that the pairing efficiency is highly sensitive to the standard deviation (SD) of the distance between the cells in the entrance channel. The pairing efficiency prediction error of our model differed by less than 5% from previous experiments. The proposed model can be used to evaluate the performance of droplet-based microfluidic devices to ensure higher precision for co-encapsulation of cells.

## 1. Introduction

Cell confinement in nano- or picoliter volumes using microdroplets has gained significant attention in recent years, especially in biosensing. Their simplicity, high throughput, and monodispersity [1,2] make droplet-based microfluidics systems suitable for biosensing [3,4], biological encapsulation [5], tissue engineering [6], spheroid formation for preclinical drug testing [7,8,9], and other applications [10,11,12,13,14,15]. Despite the vast potential, it has been shown that these systems have several limitations, including encapsulation deficiencies (heterogeneity in the number of cells per droplet) [16]. This is because the Poisson distribution determines the scattering of cells in a suspension, i.e., the randomness in the spatial cell scattering leads to low efficiency. Cells are not dispersed in an arranged sequence, but one can assume that the suspension is divided into small parts, each with a volume equal to that of the resulting droplet. The suspension concentration can be defined as the average number of cells per droplet volume. According to the Poisson distribution, the encapsulation probability of one cell in a droplet is only 37% when the concentration of the initial cell suspension is one cell per droplet. The other 63% of droplets contain zero or more than one cell [17].

When a stream of hydrophobic phases crosses a hydrophilic stream, in the presence of hydrophobic walls this can lead to a periodic water droplet formation regime, surrounded by an oil environment with a slight diameter difference (polydispersity). The Reynolds (Re), Weber (We), and capillary (Ca) numbers regulate the droplet formation regime, depending on the geometry of the droplet formation device. Several numerical methods have been employed to evaluate the effects of each parameter on the droplet formation. One of the earliest studies on flow-focusing droplet generators was done by Tan et al. [18]. Another study used multiphase flow simulations, such as the ternary lattice Boltzmann (LBM) equation [19], to model the different regimes of the double-emulsion formation and to investigate the effects of the viscosity ratios of the fluids. The volume of fluid (VOF) method [20] has been proven to be an effective technique, which can be used to precisely predict the droplet formation regime [21,22].

Several studies have been successfully conducted, introducing promising methods for effective encapsulation, including passive and active designs [23]. Active methods utilize fluorescent microscopy [24], flow cytometry [25], microchambers of bilayered microfluidics, and integrated active valves [26], or use a laser for single-cell encapsulation [27], all of which require additional sophisticated devices. In contrast, passive designs basically use hydrodynamic forces to relocate cells in the channel cross-section before emulsification [2,28,29,30] and order them longitudinally. Some researchers have also used trapping wells [31].

Droplet formation that occurs in the dripping regime is highly monodisperse [32]. As such, this is the best choice for cell encapsulation with high efficiency. The other regimes impose randomness that hampers the periodicity of encapsulation. Therefore, by confining cells in equally spaced trains behind the injection tip of a dripping site using passive methods, the number of unwanted droplets (those with zero or more than one cell) could be reduced to zero [28]. Passive approaches in single-cell encapsulation have led to encapsulation efficiencies as high as 99%, as compared to 70% in co-encapsulation [30]. If the particles entering the droplet formation site were not controlled, the probability of co-encapsulation of precisely one of each cell type would be reduced to 13.5% (37% × 37%) [2].

There are several numerical methods that have been proposed to precisely predict the migration trajectory of the cells. Liu et al. [33] suggested a four-term fitting formula to estimate the inertial lift forces exerted on particles by the fluid. This formula was obtained using the direct numerical simulation of particle motion in a straight channel. Another reliable method is DiCarlo’s [34] algorithm of computing the lift forces of the particles in straight channels, which was later modified by Martel and Toner [35] to be applied in spiral microchannels. In addition to lateral repositioning, inertial focusing devices are capable of placing cells longitudinally in a controlled order [36]. Russom et al. [37] used a curved microchannel to separate 7, 10, and 15 μm particles. They reported trains of evenly spaced particles of different sizes after passing through the curved channel.

Single- and double-cell encapsulations methods have been the subject of many experimental investigations, involving inefficient encapsulation regimes. However, to the best of our knowledge, no numerical study has systematically investigated the effects of different parameters during the whole process of cell encapsulation. Herein, a thorough computational fluid dynamic (CFD) simulation is conducted to calculate the cell pairing efficiency of a microfluidic-based droplet device. Combining the level set method with large particle tracking modeling (compared to channel dimensions), we developed a novel CFD model to capture droplet formation and cell tracking into the droplets. The modified iterative algorithm introduced by Di Carlo [34] was used to assess the body forces on the cells at different cross-sections along the path of the cells. The encapsulation efficiency was reported by counting the contents of the first 20 droplets, which was validated against the experimental data from [2]. The effects of the droplet formation frequency and standard deviation (SD) on the performance of the microfluidics device were also evaluated.

## 2. Model Description and Methods

Two different kinds of cells dispersed in an aqueous phase were co-encapsulated in droplets formed in the microfluidic platform. The cells were simplified as solid rotating particles with a constant diameter. Droplets were formed by the interaction of this aqueous phase (water) with the hydrophobic medium (oil). The volume flow rate of the medium was adjusted to $Q_{m}=10$ μL/min in each channel and the total volume flow rate of the oil phase was 45 μL/min, which was divided equally between the two oil entries ($Q_{o}=22.5$ μL/min). The physical properties of the two immiscible phases are presented in Table 1. Both particles have the same properties, however from different paths.

**Figure 1:**
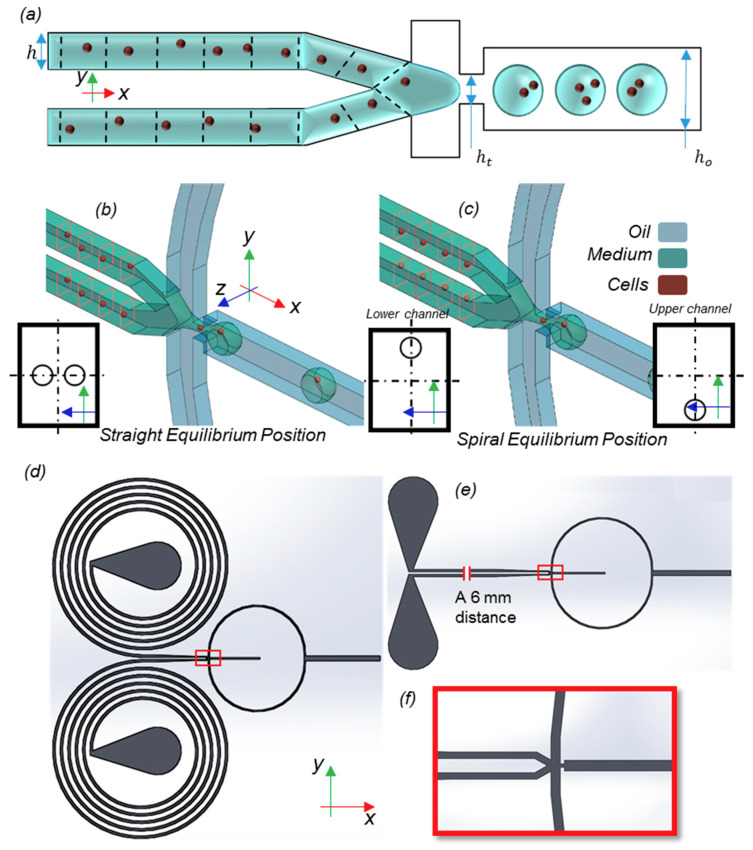
(**a**) Model and dimensions. Inertial focusing devices are used to control the injection of particles into the computational domain. (**b**,**c**) The lateral equilibrium locations of the particles in straight and spiral flow-focusing devices, respectively. In spiral microchannels with specific aspect ratios and particle densities, the equilibrium location of the particles changes to near the outer side of the spiral. The rectangular red frames show the cross-sections in which particles lie to better emphasize their local positions. (**d**) Microchannels with spiral and (**e**) straight flow-focusing devices. The magnified part (**f**) shows the droplet formation junction, which is separated with a red rectangular frame in (**d**,**e**).

The size of the particles was assumed to be the same as described in [2], in which cells were considered to be the male and female versions of special algae. Each particle type came from an inertial focusing device, with which particles were arranged into two queues, with specific randomness of the distance between the particles in each array (Figure 1a). The height (*h*) and the width (*w*) of the incoming channel were 44 and 31 μm (perpendicular to the picture), respectively. The focusing throat height was *h_t_* = 44 μm and the height of the output channel was *h_o_*. The ratio of *h_o_* to *h_t_* is called the geometry expansion factor (EF). Based on these dimensions and the definition of We and Ca numbers, $We_{m}=\frac{\rho_{m}V_{m}^{2}D_{h}}{\sigma }$ and $Ca_{o}=\frac{\mu_{o}V_{o}}{\sigma }$, in which ρ, μ, and σ are the density, viscosity, and surface tension, respectively. Subscript *m* refers to the medium and *o* denotes the oil phase. Here, V is the mean velocity in the throat and $D_{h}$ is the hydraulic diameter.

$$V_{m}=\frac{2Q_{m}}{wh_{t}},\;V_{o}=\frac{2Q_{o}}{wh_{t}},\;D_{h}=\frac{2wh_{t}}{w+h_{t}}$$

In this simulation, $We_{m}=0.0504$ and $Ca_{o}=0.0065$; both values were far less than 1 in the ensuing dripping regime. 

The flow rates of the medium and oil entries are dependent on the concentration of the cells, meaning that the frequency of the droplet formation (f) is synced with the initial cell concentration (λ). The effective diameter of the droplet (D) is correlated with λ in $=\frac{N}{\frac{\pi }{6}D^{3}}$, in which N denotes the number of cell pairs in one droplet, whereby in our case N=1.

There are several extensive numerical and experimental studies that have proposed a correlation between f, $Re_{m}=\frac{\rho_{m}V_{m}D_{h}}{\mu }$, and $Ca_{o}$ [3,18,21,38]; that is, $f=f\left(Re_{m},Ca_{o}\right)$. We know that $Q_{m}=f\times \frac{\pi }{6}D^{3}$. Using a simple rearrangement between these equations, we would get the following characteristic encapsulation equation:

$$f\left(Re_{m},Ca_{o}\;\right)=\lambda Q_{m}$$

Mathematically speaking,  f is a monotonically increasing function of $Ca_{o}$ and the right-hand side of the above expression is constant. $Re_{m}$ and $Ca_{o}$ also have narrow applicable ranges (stable dripping regime). Hence, given the values of λ and $Re_{m}$, the abovementioned equation has only one physical solution for $Q_{o}$ and $Q_{m}$. Practically, for each λ there is only one value for $Q_{m}$ and $Q_{o}$, which will lead to potentially perfect co-encapsulation.

It is computationally costly to simulate both the inertial focusing device and droplet formation parts in one simulation. Moreover, the output of the former is highly random, except for the equilibrium locations of the particles. Hence, only the droplet formation part needs to be simulated within our model. It is assumed that the particles are injected into the computational domain at specified periodic times and two specific equilibrium locations in each channel of the particle arrays. The schematic equilibrium location of the particles in each type is depicted in Figure 1b,c for a better comparison. The dimensions of the straight microchannel were chosen to be 44 μm × 31 μm as in [2]. For the spiral channel, the dimensions were chosen as 31 μm × 180 μm, so that the equilibrium positions of 10 μm particles changed to near the outer side of the channel based on the design rules explained in [39].

The output lateral locations of the long straight and spiral inertial microchannels were considered as the initial positions of the injecting particles. For both the spiral and the straight channels, the droplet formation part of the device was kept unchanged. The red-bordered picture in Figure 1c illustrates this part.

Since the particle concentration (*λ*) can be controlled by simply adjusting the suspension concentration [40], the average distance between the ordered cell arrays coming out of the inertial focusing device can be controlled. In other words, the average particle injection period can be synchronized with the time of the droplet formation in order to achieve maximum efficiency. Thereby, the time between two subsequent injections of particles is assumed to be equal to the period of droplet formation.

### 2.1. Interface Tracking

COMSOL Multiphysics 5.2 commercial code was used to solve the level set model. The continuity and momentum equations were the same as Equations (1) and (2), respectively.

(1) $$\nabla .\vec{V}=0$$

(2) $$\frac{\partial \vec{V}}{\partial t}+\nabla .\left(\vec{V}\vec{V}\right)=-\frac{\vec{\nabla p}}{\rho }+\nabla .\left[\mu \left(\nabla \vec{V}+\nabla {\vec{V}}^{T}\right)\right]+\vec{F}$$

where *V* stands for the fluid velocity in the computational cell, ρ represents density, μ represents viscosity, t is time, and p denotes the pressure. In Equation (2), F is the momentum source term, which is equal to the surface tension force (*F_st_*) plus the drag force (*F_d_*) reaction of particles divided by the total volume of computational cells containing the particle ($NV_{p}$), and the reaction force of the surface tension exerted on the particles (*F_σ_*) divided by the total volume of interface cells that were touched by the particle (*NV_σ_*), which will be discussed later in Section 2.2.2.

(3) $$\vec{F}=\vec{F_{st}}+\left(\frac{\vec{F_{d}}}{NV_{p}}+\frac{\vec{F_{\sigma }}}{NV_{\sigma }}\right)$$

Here, $F_{st}$ only appears in the cells containing the interface of the fluids, and the latter term appears in the cell containing the center of the particles. $F_{st}$ in each computational cell is calculated by Equation (4), which is called the continuum surface force (CFS) method [41].

(4) $$\vec{F_{st}}=\sigma \frac{\rho \kappa \vec{\nabla \alpha }}{\frac{1}{2}\left(\rho_{m}+\rho_{o}\right)}$$

where the constants $\rho_{o}$ and $\rho_{m}$ are the densities of the oil and medium phases, respectively, and *σ* is the surface tension coefficient. Here, *α* is the volume fraction of the medium phase within the corresponding cell and κ is the interface curvature. The curvature is equal to the divergence of the normal unit vector of the interface, as shown in Equation (5):

(5) $$\kappa =\nabla .\hat{n}$$

The normal unit vector is defined in Equation (6):

(6) $$\hat{n}=\frac{\vec{\nabla \alpha }}{\left|\vec{\nabla \alpha }\right|}$$

The flow field was solved with the CFD incompressible flow method with P2–P3 spatial discretization for velocity and pressure gradients, although time was discretized with the first-order scheme; the time step size was chosen 1.0 × 10^−8^ s.

For two distinct incompressible and immiscible phases, the level set equation could be reduced to Equation (7), which is a simple transport equation of *α*.

(7) $$\frac{\partial \alpha }{\partial t}+\vec{V}.\vec{\nabla \alpha }=\gamma \vec{\nabla }.\left(\epsilon_{ls}\vec{\nabla \alpha }-\alpha \left(1-\alpha \right)\hat{n}\right)$$

Here, $\epsilon_{ls}$ and γ are equation constants, which were adjusted for each simulation to ensure consistency in the stability. After calculating the medium volume fraction, the oil volume fraction can be calculated by subtracting *α* from 1. This equation was solved explicitly after solving the flow field (continuity and momentum) using a first-order discretization scheme for *α* Then, the overall viscosity and density can be calculated using Equations (8) and (9):

(8) $$\rho =\rho_{m}\alpha +\rho_{o}\left(1-\alpha \right)$$

(9) $$\mu =\mu_{m}\alpha +\mu_{o}\left(1-\alpha \right)$$

The constant properties of medium (dispersed phase) and oil (continuous phase) are shown in Table 1.

The boundary conditions of the domain are the fully developed velocity profiles at the inlet of the microchannel and the absolute pressure at the outlet (*p* = 0 Pa). All the other boundaries were set to be impermeable walls, with a static contact angle with the shared medium–oil interface. The contact angle value affects the cells containing the interfaces near walls and determines the direction of the surface tension force, which influences the whole flow regime [42]. In Section 3.3, the effect of the wall contact angle is reviewed thoroughly.

### 2.2. Particle Tracking

Cells are assumed as rigid, circulating spherical particles. Using lift and drag forces, the equation for the motion of particles can be written with derivatives of the locations of particles with respect to time as follows:

(10) $$m_{p}\frac{d^{2}\vec{X}}{dt^{2}}=-\vec{F_{d}}+\vec{F_{l}}-\vec{F_{\sigma }}$$

(11) $$\vec{F_{d}}=3\pi \mu D_{p}\left(\frac{d\vec{X}}{dt}-{\vec{u}}_{fluid}\right)$$

These equations were solved using the particle tracing model and using the multiphysics module; the effects of these forces were exerted in fluid. The lift forces applied on particles were calculated using a modified algorithm proposed by Ghadami et al. [43]. The modification was performed to reduce the computation time, since the initial conditions of every iterative step were set from the solution of the previous step.

The algorithm calculates the lift forces on circulating spherical particles in a channel, using a preassessed intake velocity profile for the flow and a specified cross-section. There are three main steps in this procedure. First, the flow field in the channel with the cross-section and the velocity profile at the inlet is calculated. Then, a particle is placed at the middle length of the channel in the center of the cross-section. The velocity of the fluid calculated at this location in the first solution is set for the initial estimation of *U_p_* (steady particle velocity). The rotational velocities of the particle (*ω_x_*, *ω_y_*) are set by the angular velocity of the fluid in that location. The inlet and outlet velocity profiles are set as *U_p_*–*U_xy_*_,_ and the walls are assumed to have a velocity of −*U_p_* The fluid flow is then solved for this configuration in order to calculate the net reaction forces and torques on the particles from the fluid. If the values meet the following criteria (Equation (12)), the internal loop is considered to be converged, and the values of forces and torques are stored:

(12) $$\{\begin{array}{c} \left|F_{z}\right|<1.5\times 10^{-13}\left(N\right)\\ \left|T_{x}\right|<1.0\times 10^{-20}\left(N.m\right)\\ \left|T_{y}\right|<1.0\times 10^{-20}\left(N.m\right) \end{array}$$

**Figure 2:**
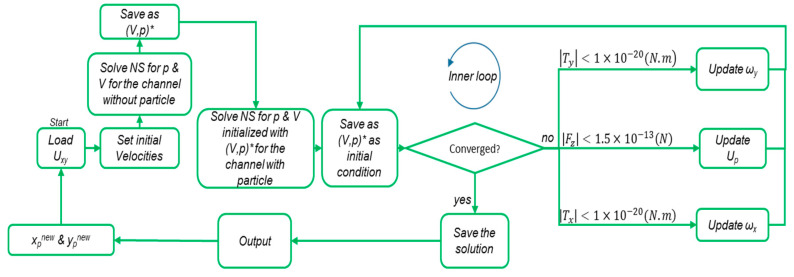
The modified algorithm for calculating the lift force exerted on particles in the entrance channels. The initial values of the *U_p_* and *ω_x_*, *ω_y_* are set from the velocity and angular velocity by solving the Navier–Stokes equations (*NS*) for laminar incompressible flow in a channel without particles. With these initial conditions, the *NS* will be solved in a channel with particles in the *x_p_* and *y_p_* positions. In the inner loop, after each iteration, the values of (*V*,*p*) are used as the initial conditions for the next iteration.

However, if the conditions are not met, new values for *U_p_* are estimated based on Equation (13):

(13) $$\{\begin{array}{c} U_{p}^{new}=U_{p}^{old}+\frac{F_{z}\Delta t}{m_{p}}\\ \omega_{x}^{new}=\omega_{x}^{old}+\frac{T_{x}\Delta t}{I_{p_{x}}}\\ \omega_{y}^{new}=\omega_{y}^{old}+\frac{T_{y}\Delta t}{I_{p_{y}}} \end{array}$$

where Δ*t* is the hypothetical time interval of the particle motion, which is a constant value and limits the convergence of the inner loop (Figure 2). The *I_px_* and *I_py_* refer to the mass inertia of the particles around the x- and y-axes, respectively (the z-axis is along the direction of flow). The inner loop continues until Equation (12) holds true and then the particle is situated in another location. These steps are repeated for the entire network of discrete locations in the cross-section.

**Figure 3:**
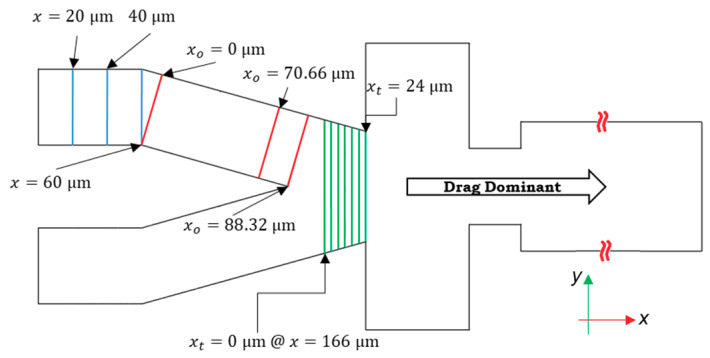
The 12 cross-sections along the length of the channel are used to calculate the lift forces on particles considering trilinear interpolation between these sections. Particles are dominated by the drag force in the right portion of the design after the tapered section; therefore, the effect of lift forces on particle encapsulation is negligible in this zone. Here, $x_{o}$ stands for the local *x*-coordinate laid in the direction of the oriented channel, and subscript *t* shows the local x-coordinate of the tapered section.

Because the velocity profile and the shape of the channel vary at different cross-sections through the channel length, trilinear interpolation is used to evaluate lift force at any point. There are 12 cross-sections in the channel, in which the calculations of lift forces are performed (Figure 3). These 12 cross-sections are chosen based on the SD of the difference between the velocity profiles in the non-dimensionalized cross-sections. In other words, if the SD is higher than 0.001 m/s, the section is chosen for calculations, and if not the trilinear interpolation is used.

Since the flow rate and the shape of both cell channels are the same and the two cell types are of equal size, the lift forces on both cell types in the channel have the same magnitude.

The flow profiles in every cross-section are symmetrical with respect to the xy-plane in the global coordinate. The code for calculating the lift forces is used for the points in the right side of the cross-sections. However, on the other side, the symmetry condition is implemented. The lift force in the direction of y (*F_y_*) in the local coordinates of the cross-sections does not vary between these halves, but (*F_x_*) is in the opposite direction with equal magnitude. After the cross-section at *x* = 190 μm (locally *x_t_* = 24 μm), because two streams of the flow intersect, the maximum velocity increases. Based on Equation (14), as the difference between the particle and fluid velocity increases, the magnitude of *F_d_* increases. We assume that after this cross-section, only the drag and interface reaction control the particles’ trajectory.

(14) $$\vec{F_{l}}=\{\begin{array}{c} 0;\\ interpolation; \end{array}\begin{array}{c} x>190\;\mu m\\ x\leq 190\;\mu m \end{array}$$

A MATLAB R2018a code was developed in COMSOL with MATLAB module to implement the loop in Figure 2 to calculate the lift forces. After complete calculation of the lift forces in each cross-section, the interpolation function in COMSOL was used to calculate the forces in each point in the 3D space of the computational domain.

#### 2.2.1. Gaussian Random Number Generator (GRNG)

This model uses Gaussian distribution with a specified SD and a mean value to calculate the random location of each particle at the injection time. The Gaussian random number generator (GRNG) determines the horizontal injection locations of particles with a prespecified SD from the reference points, i.e., (*x* = 0 μm). Therefore, the time interval between two successive injections does not vary, but the location differs with respect to the SD.

#### 2.2.2. Interaction of Particles with the Interface of Water in Oil (W/O) Droplets

**Figure 4:**
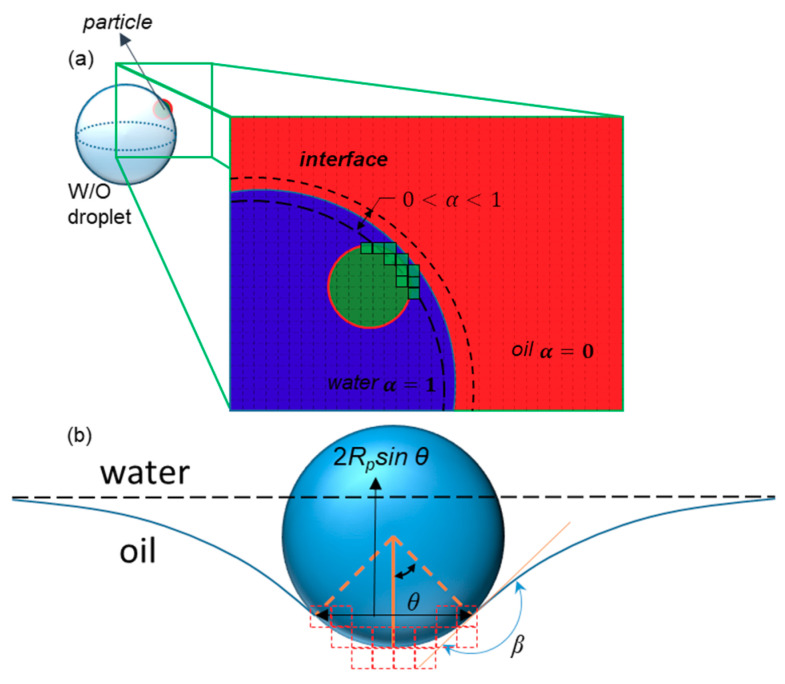
The interaction between a particle and an interface. (**a**) Green cells are those with volume fractions between 0 and 1 that are touched by the particle, or those for which the distance between their center and the center of the particle is less than the particle radius. The total volume of these cells equals *NV_σ_*. (**b**) The dimensions and parameters used to calculate the surface tension force exerted on the particle by the interface. The dashed red lines show the faces of touched cells.

The drag and lift forces in the Lagrangian model, in which all particles are considered as small dots, could be ignored when implementing the interactive force at the particle–fluid interface. To take the interactions between the interfaces of the droplets and particles into account, we used a code to impose the interface–particle interaction. This code checks whether a particle meets the interface and then uses the surface tension to exert force on the particles, i.e., *F_σ_* in Equation (15) by ignoring the gravity term [44]. Additionally, the drag force exerted on particles is added by Stokes assumption (Equation (11)). The direction of this force is perpendicular to the interface of the fluid, which is in the opposite direction of the normal vector calculated by Equation (6). The *θ* in this equation is shown in Figure 4b, *R_p_* is the radius of the particle, and σ is the coefficient of the surface tension:

(15) $$F_{\sigma }=2\pi \sigma R_{p}sin\theta sin\beta$$

In which *β* is the constant contact angle of the particle with the interface. In the discretized domain (Figure 4a), the term sin *θ* is calculated by finding the maximum distance between the touched cells, which is attributed to $2R_{p}sin\theta$, as shown in Figure 4b. On the other hand, when the particle crosses the interface, the force slows it down. The particles in our simulation are assumed to be hydrophilic (*β* = 150°).

## 3. Results and Discussion

In this section, the results of lift forces, simulations of droplet formation, and particle encapsulation are discussed in detail. The verification of the model itself in terms of grid dependency is presented. The pairing efficiency result is in good agreement with the experimental results in [2]. First, the lift force vectors are displayed in different cross-sections along the channel. Secondly, the diameter and frequency of the droplet formation are further explained based on quantitative and qualitative results, and finally the efficacy of encapsulation and the effects of frequency and randomness of scattering on the efficiency are demonstrated.

### 3.1. Grid Study and Time Independency

**Figure 5:**
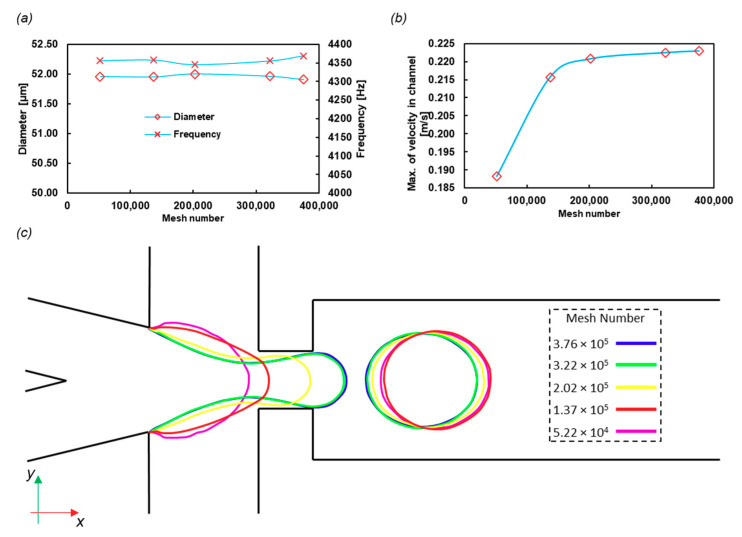
Grid study on (**a**) the droplet diameter and frequency and (**b**) the velocity of the center of the incoming channel. The mesh number effect on the droplet diameter is not significant (less than 1%), and thus the lowest number of mesh cells needed for calculations is determined by other variables, such as the velocity field. (**c**) The W/O interface in the midplane of the domain in *t* = 0.0005 s with different mesh numbers, which shows the independence of the mesh.

The final frequency of the droplet formation and the diameters of the droplets were calculated with different mesh numbers. The results are shown in Figure 5a, and the structured meshes used in this part are shown in Figure S1. The frequency and diameter variations with mesh size are negligible. However, Figure 5b indicates that the fluid velocity in the middle of the incoming channel varies with the mesh number, although it reaches a constant value after the 3 × 10^5^ mesh number. The number of meshes was chosen near 4 × 10^5^ as a conservative approach.

To check the time independency, we chose the Courant number equal to 0.3 to run the code for different mesh numbers. The results of the interface of water and oil at the same time (*t* = 0.0005 s) are shown in Figure 5c. The interface for the last two large meshes is acceptably overlapped.

### 3.2. Lift Forces

**Figure 6:**
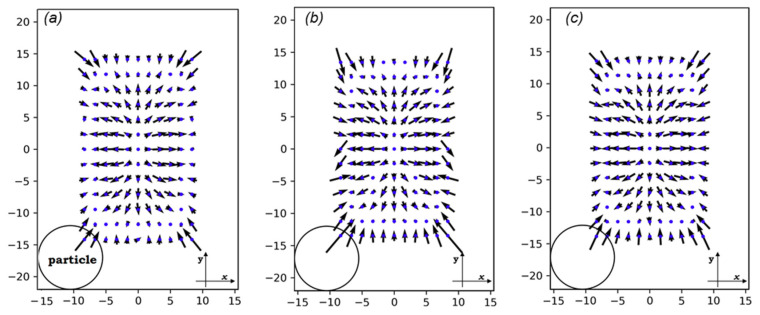
The lift forces on three different cross-sections: (**a**) on *x* = 20 μm, (**b**) on *x* = 0, and (**c**) on *x*_0_ = 88.32 μm (see Figure 3). The x-y coordinate is a local coordinate whose dimensions are shown on each rectangle. The circle only exhibits the scale of the particle dimensions in comparison with those of the channel. All dimensions are in μm.

Figure 6 shows the vectors of planar forces exerted on particles as they pass through the cross-sections. At the very center of every cross-section, no force is applied on the particle. However, when it slightly deviates from the center, the fluid pushes it towards the walls. Near the walls and the corners, the magnitude of the lift forces dramatically increases. This is because of the increased velocity of the fluid between the wall and particle compared to when there are no particles in the channel. The direction of forces near the walls is so that particles are pushed to the center.

The magnitude of the lift forces depends on the velocity in the channel cross-section and the cell size. If the size of the cells is small (<10 μm) compared to the channel dimensions, the inertial focusing platforms will not be not effective enough in ordering them, leading to low encapsulation efficiency [2]. For cell sizes close to the channel dimensions, the risk of clogging will increase [45]. Furthermore, the high shear rates at the intersection of the oil and medium channels in such conditions would deform cells and bring about unavoidable damage to cells in large droplet formation frequencies. Therefore, a cell size of 10 μm is a valid choice for our case. In this range, lift forces enforce cell migration to x=±8 μm and y=0 μm in the cross-section’s local coordinates.

### 3.3. Droplet Formation

**Figure 7:**
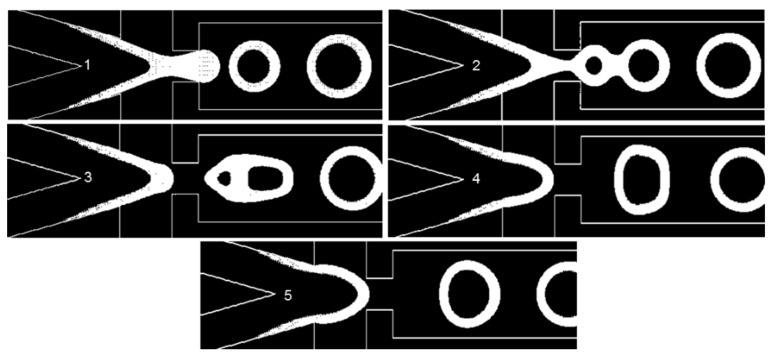
Here, *θ_wall_* = 5°, *σ* = 0.06 N/m, and EF = 2.63. The pictures numbered 1 to 5 show the W/O common interface at different time instants in the injection tip (Video S1).

It is important to have a periodic regime of droplet formation that has few variations between different periods to increase the efficiency of encapsulation. We call this state of little variation a stable regime (See Supplementary Materials Figure S2). Nevertheless, some physical and geometrical parameters will cause this regime to undergo variations, which we call instabilities. Another form of instability in the dripping regime is inherent to flow-focusing droplet generators [38]. One example of such instability is shown in Figure 7 and Video S1. When the second droplet is forming, the distance between their surfaces will cause a fusion. This fusion changes the downstream velocity and pressure contours significantly and impairs the stability. The contact angle ($\theta_{wall}$), surface tension, and EF will affect the stability of the dripping regime, as they are physical and geometrical parameters. Another source of instability is when a droplet reaches the outlet. Due to the pressure outlet boundary condition, a vacuum is imposed in the droplet, causing artificial suction. The length of the downstream area of the microfluidics system is assumed to be 2 mm, in order to avoid any droplet reaching the outlet whilst the new droplets are forming (Video S2). 

Here, the effects of the wall contact angle and the coefficient of the surface tension on the stability of the droplet formation and the geometrical EF are investigated. The *θ_wall_* was considered in the range of 5° (superhydrophobic) to 75°, and σ in the range of 0.05207–0.06 N/m. Two expansion factors of 1.71 and 2.63 were also simulated to calculate the first 10 droplet diameters to investigate the stability.

**Figure 8:**
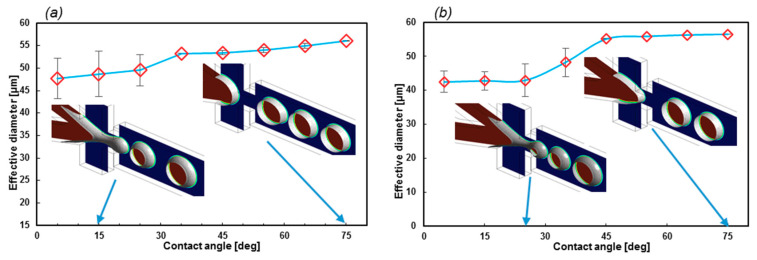
(**a**) Here, *σ* = 0.06 and (**b**) *σ* = 0.05207 N/m for EF = 2.63. The average droplet diameter for the first 10 droplets. The superhydrophobic wall contact angles produce a less stable regime. The red color shows the medium phase.

Figure 8 reveals that even though the contact angle can be used to control the droplet diameter, the stability of the droplet formation dramatically collapses when *θ_wall_* decreases. However, at higher contact angles, the variations in the droplet diameters are negligible. Moreover, reducing *σ* results in a less stable regime, as can be understood from regions of stability. The stable region for *σ* = 0.06 N/m is 35° to 75°, while it is 45° to 75° for *σ* = 0.05207 N/m. More simulations of lower surface tension coefficients led to unstable regimes at all contact angles.

**Figure 9:**
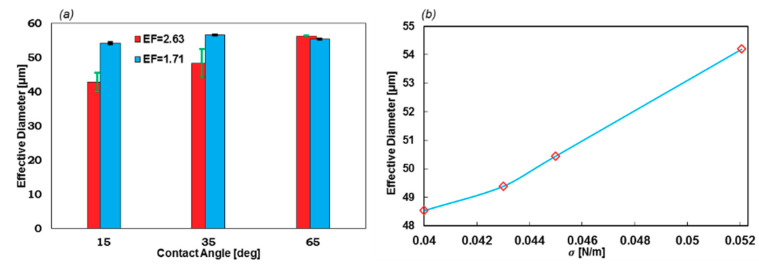
(**a**) The effect of the geometrical EF on the droplet formation stability and size with respect to different contact angles (*σ* = 0.05207 N/m]) and (**b**) droplet diameters with respect to *σ*. The greater the surface tension coefficient, the larger the droplets.

EF is also a significant factor in the geometry design of the formation tip. As Figure 9a suggests, lower EF leads to a stable regime with a narrow error bar. On the contrary, with higher EF, the error bars become larger. Furthermore, the droplet diameter in stable regimes (*θ_wall_* = 65°) does not change with EF. Moreover, the droplets with lower EF are uniform at different contact angles, which indicates that if the constant wall contact angle cannot be applied as the boundary condition, this geometry will generate droplets with high uniformity.

The effect of the σ on the EF = 1.71 and *θ_wall_* = 15°, which is illustrated in Figure 9b. By increasing the value of *σ*, the droplet diameter will increase. Meanwhile, the stability is not subjected to change in this chart.

### 3.4. Encapsulation

For a 1:1 ratio of the number of type A particles to the number of type B particles in a droplet, the flow rate of the suspensions in both channels are assumed to be equal. Hence, half of the droplet volume comes from the upper channel and the rest from the lower channel. This amount of fluid occupies the length of the incoming channel before being combined with the other half and broken into the droplet. This length is referred to as X. If the distance between the particles in the channel is exactly equal to X, all the droplets must have a 1:1 ratio. However, as has been seen from the experiments [29], this parameter has random scattering of the specific SD and mean value. The mean value should be equal to X. However, the SD varies depending on the efficacy of the inertial focusing device.

Because of the presence of the lift forces in the channels, the lateral equilibrium locations of the particles in the incoming channels are well known. Therefore, after a long straight or spiral path, it is an acceptable assumption that the locations of the particles in y and z directions (Figure 1) are the equilibrium locations. Nevertheless, the x locations of particles do not have any mechanisms to bring themselves to a specified x-location with respect to the other particles, other than the mere two-way effect of particles on the flow of the particles. The so-called “two-way interaction” arises from the x-velocity difference between particles and flow. After a considerable distance passing through the focusing channel, particles will reach a steady velocity at their lateral position. Hence, the SD of the x-location is the priority. One can show that any perturbations in the y and z locations of particles from equilibrium locations would be dampened in a matter of milliseconds (based on the relaxation time of the Stokes drag and the lift forces), but the particle would end up in another x location compared to it started from the equilibrium position. This is because of the difference in the medium velocity profile in different y and z locations. This means that x-location variation is a lot more sensitive to any noise or variation from the surroundings.

**Figure 10:**
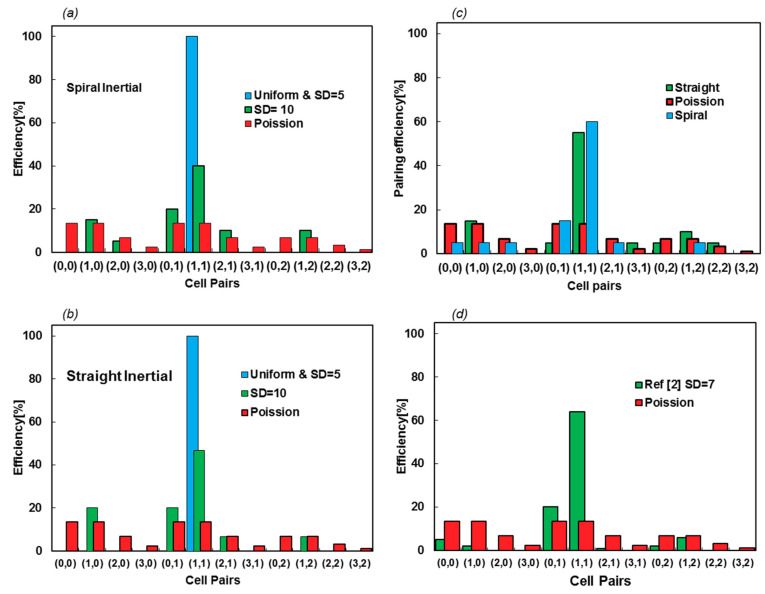
(**a**,**b**) Encapsulation efficiency of spiral and straight inertial focusing devices for two SD values of 5 and 10 μm. The scattering of the droplet contents shows similar productivity for both devices; other than small variations in the cell pair scattering, changing the focusing channel type does not affect the efficacy of the encapsulation. (**c**) The pairing efficiency increases by lowering the frequency. The frequency is reduced by increasing *σ* to 0.065 N/m. (**d**) The efficiency values and scattering cell pairs are from [2], with an approximate SD of 7 μm.

Here, simulations are performed for the uniform distribution, while SDs of 5 and 10 μm with a frequency of 5 kHz and the efficiency are evaluated, counting the contents of 20 microcapsules. The numbers of particles in droplets are enumerated, as they are encapsulated in the droplets. The first and second numbers in each pair refer to the numbers of type A and B particles, respectively. Figure 10a,b show the efficiency of the encapsulated pairs for two types of inertial focusing devices. The Poisson scattering is also assessed to display the difference between the fully random injection and the impacts of using focusing devices.

The efficiency of encapsulation is 100% for SD = 5 μm, which can be seen in Videos S3 and S4 for straight and spiral inputs, respectively. Nevertheless, this decreases to about 45% for SD = 10 μm (Videos S5 and S6). This shows that the efficiency of encapsulation is highly sensitive to particle scattering and the function of the inertial focusing device. Figures S3 and S4 show the space–time diagrams of cells in conjunction with the droplet formation time, accentuating how SD affects the resulting efficiency from a synchronization point of view. More interestingly, the injection locations of either spiral or straight inertial devices cannot affect the efficacy of the device, as the pairing efficiency does not vary between spiral and straight focusing microfluidics device. Figure 10c shows how reducing the frequency from 5 kHz to 3.3 kHz increases the efficiency for SD = 10 μm. For this case, the particle concentration is reduced, such that X matches that frequency.

This model predicts 47% and 100% pairing efficiency for SD = 10 and 5 μm, respectively, as shown in Figure 10b. Based on the supplementary information in [2], the SD of entering particles is approximately 7 μm, while the efficiency is reported to be 64%. Linear interpolation between the two simulated cases leads to 68.2% encapsulation efficiency, which has less than 5 percent error. Now, it is possible to exploit our model to evaluate the design or property of the fluids. In Section 3.4, the effect of surface tension is evaluated as an example.

## 4. Conclusions

Herein, a novel method for tracking particles through a microchannel for droplet microfluidics was developed to calculate the encapsulation efficiency. The model had two parts, namely the droplet formation and particle tracking, using the level set method and particle tracing in COMSOL Multiphysics 5.2, respectively. The interaction at the particle–droplet interface was taken into consideration. The combination of the tracking of large particles in microchannels and interface tracking had not been considered in any computational works in the literature. The lift forces on particles were calculated by direct numerical simulation of the flow around particles, and no other model was used. Instabilities in the droplet formation regime strongly affected the efficiency of the encapsulation. The effects of surface tension coefficient, wall contact angle, and expansion factor (EF) of the flow-focusing geometry on the stability of the droplet formation were investigated numerically. The encapsulation results of our model agreed well with those of previous experimental works. The paring efficiency of SD = 7 μm was reported to be 64%; considering SD = 5 and 10 μm and assuming linear interpolation between these two points, the model prediction efficiency was 68.2%, which did not differ by more than 5% from the experimental results.

The sensitivity of particle scattering randomness on the efficiency of encapsulation was obtained for two different injection locations of particles. One was related to a spiral inertial focusing microfluidics device, and the other was the equilibrium location of particles in the output of a straight microchannel. It was shown that this location did not significantly affect the pairing efficiency. However, the sensitivity of the encapsulation efficiency to randomness in the cell queue was significantly high, suggesting more focus is needed on the efficiency of the inertial focusing device in the case of high-frequency co-encapsulation. Finally, we foresee the application of our model in evaluating the accuracy of any microfluidic device designed for encapsulation in biosensing, and also in fundamental studies of multiphase fluid and particle interactions.

## Figures and Tables

**Table 1:** Physical properties of the medium and oil.

Item/Properties	Density (kg/m^3^)	Viscosity (Pa.s)	Surface Tension (N/m)
Entry 1 Medium	998.2	1.003 × 10^−3^	0.043
Entry 2 Oil	866.0	5.076 × 10^−4^	

## Supplementary Materials

The following are available online at https://www.mdpi.com/1424-8220/20/13/3774/s1. Figure S1: The meshes used for calculations in the midplane of the 3D domain. Figure S2: The variation between droplet diameters over 10 generation for $\sigma =0.043\frac{N}{m}$ and $\theta_{wall}=75{}^{\circ}$ in EF = 1.71. Once the effect of initial condition is damped, the variation in droplet generation regime becomes minimum and dripping regimes becomes stable. Figure S3. (a) Space-Time diagram of particles in the case of SD = 0 μm, straight channel and EF = 1.71. First two droplets are forming while there is no cell available in the site of formation. (b) Maximum velocity of the whole domain which represents the time of droplet generation. (dashed green lines in time). If the maximum velocity of the whole domain is greater 4.5 m/s as a peak it will show the droplet generation. By the time that droplet is forming, if the cells x-location is greater than 270 μm then they would end up in a droplet and be encapsulated. Blue and red balls on the top of the diagram show the resulting pair of the cells. Figure S4. (a) Space-Time diagram of particles in the case of SD = 10 μm, spiral channel and EF = 1.71. First two droplets are forming while there is no cell available in the site of formation. (b) Maximum velocity of the whole domain which represents the time of droplet generation. (dashed green lines in time). If the maximum velocity of the whole domain is greater 4.5 m/s as a peak it will show the droplet generation. By the time that droplet is forming, if the cells x-location is greater than 270 μm then they would end up in a droplet and be encapsulated. Blue and red balls on the top of the diagram show the resulting pair of the cells. Video S1: Unstable regime producing non-uniform droplets. Video S2: Increasing the downstream length to suppress the effect of constant pressure at the outlet. Video S3: Efficiency of encapsulation is 100% for SD = 5 μm. Video S4: Efficiency of encapsulation is 100% for SD = 5 μm. Video S5: Encapsulation efficiency decreases to 45% for SD = 10 μm for spiral input. Video S6: Encapsulation for straight input of particles with SD = 10 μm.
