# Supplementary material for: An Interface–Particle Interaction Approach for Evaluation of the Co-Encapsulation Efficiency of Cells in a Flow-Focusing Droplet Generator
Source: Sensors (Basel). 2020 Jul 5;20(13):3774. doi: 10.3390/s20133774 (PMC7374427; doi:10.3390/s20133774)
Supplement: Supplementary file 1 [file sensors-20-03774-s001.zip › sensors-818226 supplementary/supplementary.docx]

Article

An Interface–Particle Interaction Approach for Evaluation of the Co-Encapsulation Efficiency of Cells in a Flow-Focusing Droplet Generator

Mohammad Yaghoobi ^1^, Mohammad Said Saidi ^1,*^, Sepehr Ghadami ^2^ and
Navid Kashaninejad ^3,*^

^1^ Department of Mechanical Engineering, Sharif University of Technology, Azadi St., Tehran 1136511155, Iran; mhmd.jcb1993@gmail.com

^2^ Department of Mechanical Engineering, University of Waterloo, 200 University Avenue West, N2L 3G, Waterloo, ON N2L 3G1, Canada; sghadami@uwaterloo.ca

^3^ Queensland Micro- and Nanotechnology Centre, Nathan Campus, Griffith University, 170 Kessels Road, Brisbane QLD 4111, Australia

***** Correspondence: [mssaidi@sharif.edu](mailto:mssaidi@sharif.edu) (M.S.S.) and [n.kashaninejad@griffith.edu.au](javascript:void(0);) (N.K.)

Received: 13 May 2020; Accepted: 1 July 2020; Published: date

**Structured Mesh**

The mesh independency evaluated in the paper is performed using structured meshes which last four are shown in the Figure S1 (a–d).


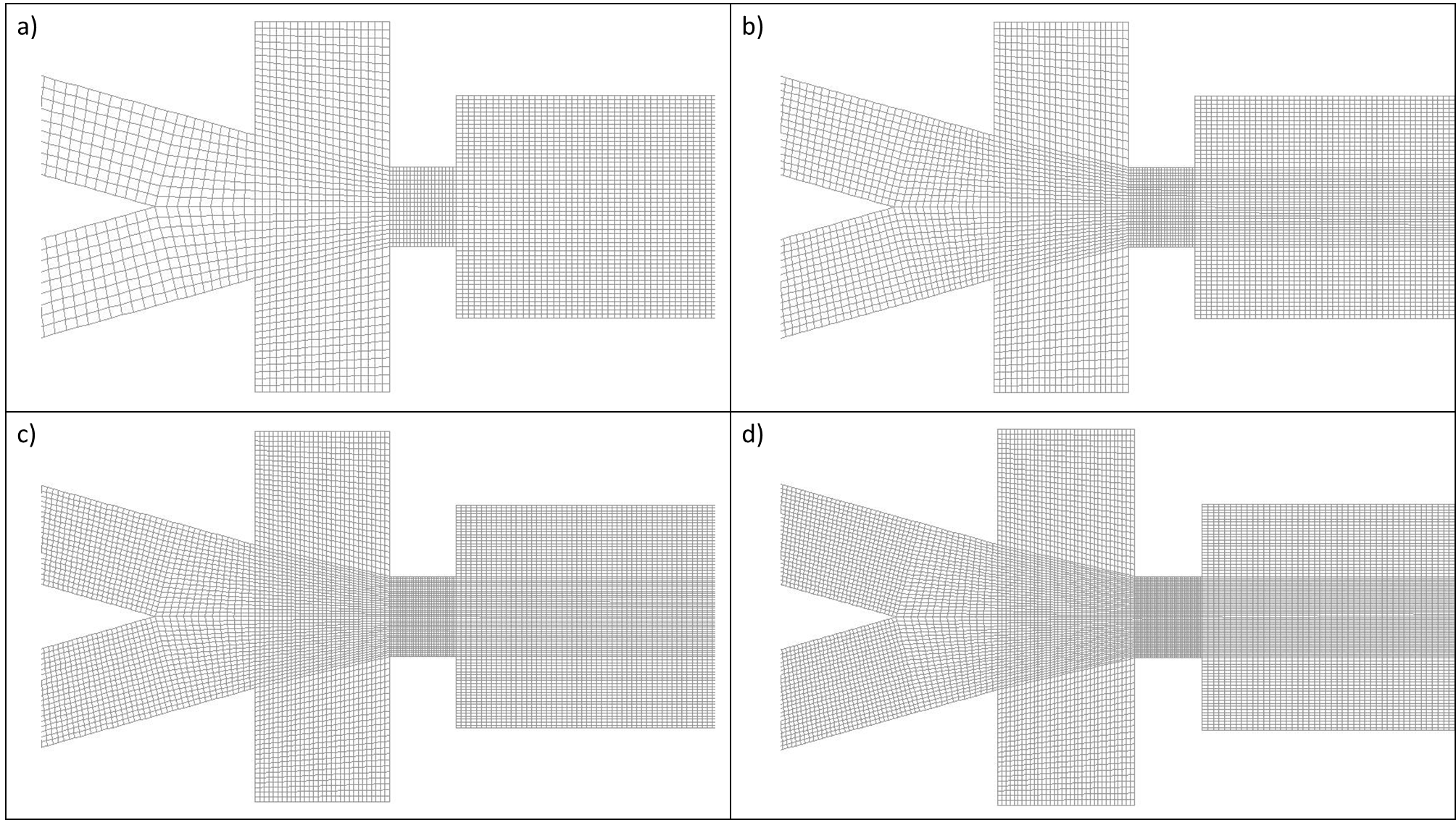


**Figure S1.** The meshes used for calculations in the midplane of the 3d domain from (a) 1.37 × 10^5^,
(b) 2.02 × 10^5^, (c) 3.22 × 10^5^ and (d) 3.76×10^5^ meshes for the geometry that has not the downstream thread of the channel.

**Stability in the droplet formation**

When the first droplets are forming the downstream of the flow is empty and also effect of initial condition is not yet damped therefore we see some numerical variation in the droplet diameter. In the Figure S4 the variation between droplet diameter over droplet generation number.


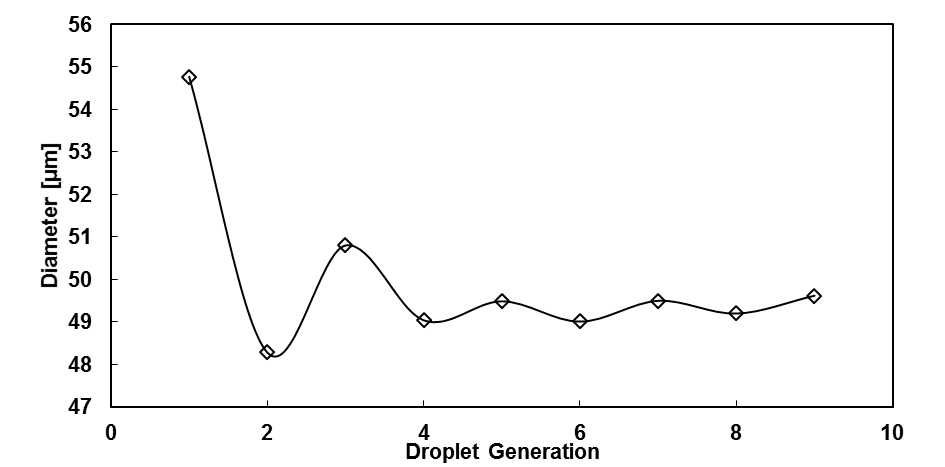


**Figure S2.** The variation between droplet diameters over 10 generations for $\sigma=0.043\frac{N}{m}$ and $\theta_{wall}=75^{\circ}$ in EF=1.71. Once the effect of the initial condition is damped, the variation in droplet generation regime becomes minimum, and dripping regimes becomes stable.

However, in some range of $Re_{m}$ and $Ca_{o}$ the regime of the flow undergoes distinguishable instabilities in the dripping regime [1]; called unstable dripping, which in the case of cell encapsulation are unfavorable. In the case shown in Figure S2, the initial instabilities are damped after 4^th^ generation, and the dripping regime becomes stable. But in $\theta_{wall}=25^{\circ}$ the same case results in an unstable dripping regime.

**Space-time synchronization with droplet formation**


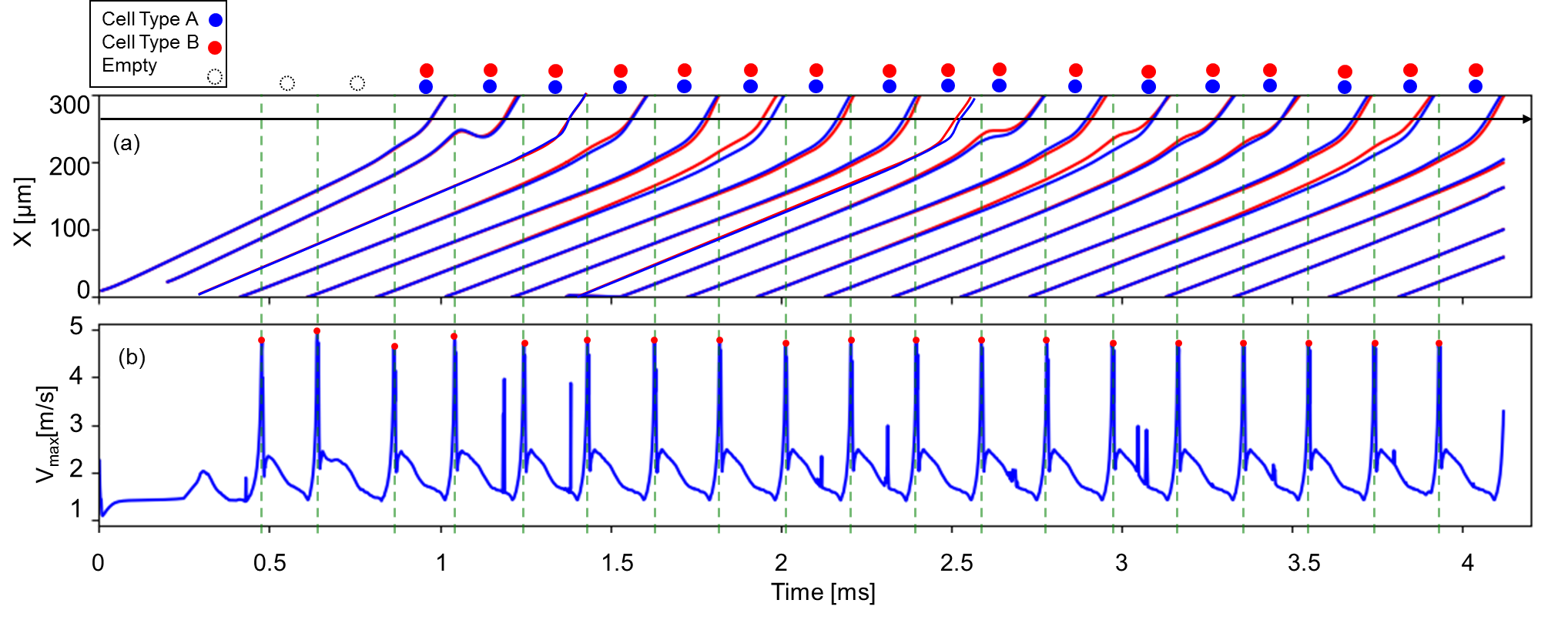


**Figure S3.** (a) Space-Time diagram of particles in the case of SD = 0 μm, straight channel and EF = 1.71. First two droplets are forming while there is no cell available in the site of formation. (b) Maximum velocity of the whole domain, which represents the time of droplet generation. (dashed green lines in time). If the maximum velocity of the whole domain is greater 4.5 m/s as a peak, it will show the droplet generation. By the time that droplet is forming, if the cells x-location is higher than 270 μm, then they would end up in a droplet and be encapsulated. Blue and red balls on the top of the diagram show the resulting pair of the cells.


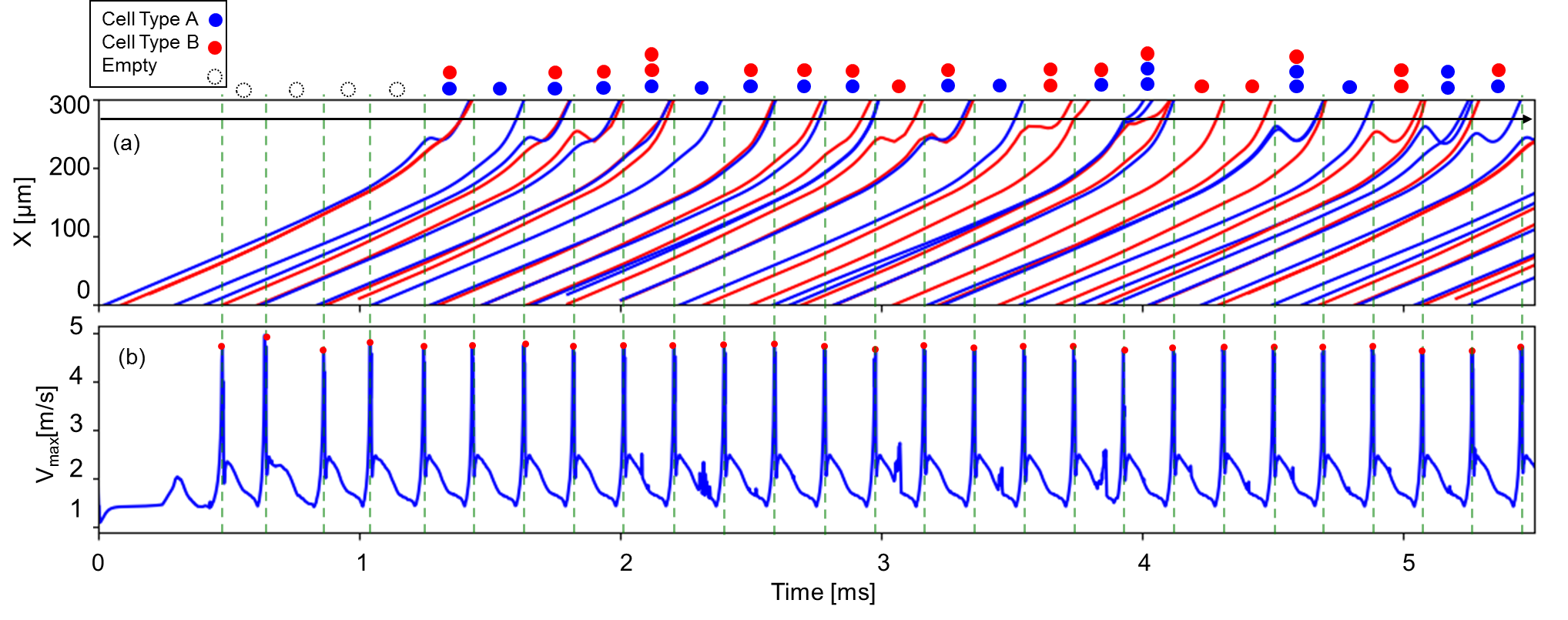


**Figure S4.** (a) Space-Time diagram of particles in the case of SD = 10 μm, spiral channel and EF = 1.71. First two droplets are forming while there is no cell available in the site of formation. (b) Maximum velocity of the whole domain, which represents the time of droplet generation. (dashed green lines in time). If the maximum velocity of the whole domain is higher 4.5 m/s as a peak, it will show the droplet generation. By the time that droplet is forming, if the cells x-location is higher than 270 μm, then they would end up in a droplet and be encapsulated. Blue and red balls on the top of the diagram show the resulting pair of the cells.

References

1. Mastiani, M.; Seo, S.; Riou, B.; Kim, M., High inertial microfluidics for droplet generation in a flow-focusing geometry. *Biomedical microdevices* **2019**, *21*, 50.
